# Gender-specific inequalities in coverage of Publicly Funded Health Insurance Schemes in Southern States of India: evidence from National Family Health Surveys

**DOI:** 10.1186/s12889-023-17231-0

**Published:** 2023-12-04

**Authors:** Santosh Kumar Sharma, Devaki Nambiar, Hari Sankar, Jaison Joseph, Surya Surendran, Gloria Benny

**Affiliations:** 1 The George Institute for Global Health, New Delhi, India; 2 Healthier Societies, The George Institute for Global Health, New Delhi, India; 3 Faculty of Medicine, University of New South Wales, Sydney, Australia; 4 Prasanna School of Public Health, Manipal Academy of Higher Education, Manipal, India

**Keywords:** Publicly funded health insurance, Gender, Inequalities, Universal health coverage

## Abstract

**Background:**

Publicly Funded Health Insurance Schemes (PFHIS) are intended to play a role in achieving Universal Health Coverage (UHC). In countries like India, PFHISs have low penetrance and provide limited coverage of services and of family members within households, which can mean that women lose out. Gender inequities in relation to financial risk protection are understudied. Given the emphasis being placed on achieving UHC for all in India, this paper examined intersecting gender inequalities and changes in PFHIS coverage in southern India, where its penetrance is greater and of longer duration.

**Data and methods:**

This study used the fourth (NFHS-4, 2015–16) and fifth (NFHS-5, 2019–21) rounds of India’s National Family Health Survey for five southern states: namely, Andhra Pradesh, Karnataka, Kerala, Tamil Nadu, and Telangana. The World Health Organization’s Health Equity Assessment Toolkit (HEAT) Plus and Stata were used to analyse PFHIS coverage disaggregated by seven dimensions of inequality. Ratios and differences for binary dimensions; Between Group Variance and Theil Index for unordered dimensions; Absolute and Relative Concentration Index (RCI) for ordered dimensions were computed separately for women and men.

**Results:**

Overall, PFHIS coverage increased significantly (*p* < 0.001) among women and men in Andhra Pradesh, and Kerala from NFHS-4 to NFHS-5. Overall, men had higher PFHIS coverage than women, especially in Andhra Pradesh, Tamil Nadu, and Telangana in both surveys. In both absolute and relative terms, PFHIS coverage was concentrated among older women and men across all states; age-related inequalities were higher among women than men in both surveys in Andhra Pradesh, Kerala, and Telengana. The magnitude of education-related inequalities was twice as high as among women in Telangana (RCI_NFHS-4_: -12.23; RCI_NFHS-5:_ -9.98) and Andhra Pradesh (RCI_NFHS-4_: -8.05; RCI_NFHS-5:_ -7.84) as compared to men in Telangana (RCI_NFHS-4_: -5.58; RCI_NFHS-5:_ -2.30) and Andhra Pradesh (RCI_NFHS-4_: -4.40; RCI_NFHS-5:_ -3.12) and these inequalities remained in NFHS-5, suggesting that lower education level women had greater coverage. In the latter survey, a high magnitude of wealth-related inequality was observed in women (RCI_NFHS-4_: -15.78; RCI_NFHS-5_: -14.36) and men (RCI_NFHS-4_: -20.42; RCI_NFHS-5_: -13.84) belonging to Kerala, whereas this inequality has decreased from NFHS-4 to NFHS-5., again suggestive of greater coverage among poorer populations. Caste-related inequalities were higher in women than men in both surveys, the magnitude of inequalities decreased between 2015–16 and 2019–20.

**Conclusions:**

We found gender inequalities in self-reported enrolment in southern states with long-standing PFHIS. Inequalities favoured the poor, uneducated and elderly, which is to some extend desirable when rolling out a PFHIS intended for harder to reach populations. However, religion and caste-based inequalities, while reducing, were still prevalent among women. If PFHIS are to truly offer financial risk protection, they must address the intersecting marginalization faced by women and men, while meeting eventual goals of risk pooling, indicated by high coverage and low inequality across population sub-groups.

**Supplementary Information:**

The online version contains supplementary material available at 10.1186/s12889-023-17231-0.

## Background

Publicly Funded Health Insurance Schemes (PFHIS) in Low and Low-to Middle-Income Countries (LICs and LMICs) are viewed as crucial components of achieving Universal Health Coverage (UHC), in that they may enhance service access and assure financial security for health service seekers [1–4]. PFHIS in India have been around for more than a decade [5–7], and are quite central in the health policy landscape presently [1]. A substantial body of evidence on the impact of PFHIS in India indicates that it has failed to achieve financial risk protection [4, 7–11]. PFHIS are associated with reductions in out-of-pocket Expenditure (OOPE) [5, 12, 13], increases in [5, 10, 11, 14] as well as stagnant utilization of hospital-care [4, 11]. In 2018, the central government launched a large PFHI programme called Ayushman Bharat Pradhan Mantri Jan Arogaya Yojana (ABPM-JAY), an expansion of the erstwhile *Rashtriya Swasthya Bima Yojna* (RSBY) and state level schemes that had been providing free hospital care [15]. The merits and demerits of the PFHI-based policy are being vigorously debated in India and also internationally [1, 16–21]. A little over two-fifths, or 41%, of households in India have at least one individual covered by a health insurance plan or health scheme, according to the recently released National Family Health Survey-5 (NFHS-5) [22], increasing from 29% in 2015–16 [23]. The fifth edition of the NFHS survey, the data of which pertains to 2019–21, shows a significant improvement in health insurance coverage in the country despite remaining far from satisfactory [22].

Large disparities in health outcomes and service coverage rates amongst population groups are evident in many countries, showing that the equity principles embedded in UHC are not being upheld [24–26]. In disaggregating health coverage data, one group which is often shown to be disadvantaged are women, who may at times in their life-cycle have greater healthcare needs than men but may often have a lower ability to pay for services [26]. According to the definition of UHC, many women ought to be the beneficiaries of cross-subsidies from more privileged groups in society in accessing health services, but this is clearly not happening at sufficient scale [24]. A substantial body of research has documented gender bias in the allocation of household resources and particularly healthcare system inputs, with adverse health outcomes for women [3, 26, 27]. Expanding access to and heavily subsidizing health care has been a key policy response to address health inequality, including inequalities in health that intersect with gender [27].

Health insurance coverage in India is fairly low. According to NFHS-5, only 30 percent of women aged 15–49 and 33 percent of men aged 15–49 are covered by health insurance or a health scheme. Almost half (46%) of those with insurance are covered by a state health insurance scheme and about one-sixth (16%) are covered by RSBY [22]. Three to six percent of women and four to seven percent of men are covered by the Employee State Insurance Scheme (ESIS) or the Central Government Health Scheme (CGHS). The highest proportion of households covered under health insurance or a health scheme in the Southern states of India is in Andhra Pradesh (80%), which launched the Rajiv Arogyasri health insurance scheme covering hospitalization expenses for secondary and tertiary care conditions through empanelled public and private hospitals in 2007 [1]. In 2008, with the introduction of RSBY, by the Central Government many states implemented this scheme following the national guidelines. Karnataka as per the latest NFHS had only 32% coverage, but actually introduced the first PFHIS scheme called Yeshasvini Corporative Farmers Health Care Insurance in 2003. This scheme offers cashless hospitalization for members and families of the co-operative societies through empaneled hospitals. 823 surgical procedures are covered and scheme offers free medical surgery worth Rs. 1.25 Lakh to Rs. 2 Lakh for its rural members and up to Rs. 1.75 Lakh for one-time hospitalisation to its urban members. For multiple hospitalisations, individuals covered under this scheme can claim up to Rs. 2.50 Lakh [28, 29].Karnataka rolled out Vajpayee Arogyasri scheme in 2009 covering hospitalization expenses for cardiac, cancer, neurological and paediatric conditions for Below Poverty Line (BPL) population. Kerala in 2008, implemented RSBY across all 14 districts covering the BPL families as per central list and later introduced the Comprehensive Health Insurance Scheme (CHIS) by expanding the beneficiary base, covering the BPL families as per state list. Tamil Nadu in 2009, implemented the Chief Minister Comprehensive Health Insurance Scheme (Kalaingar Kappittu Thittam) to provide cashless hospitalization of 1090 procedures with a coverage of INR 5 Lakh per family [1, 30]. After the rollout of AB-PMJAY many states like Karnataka, Kerala, Tamil Nadu have converged the risk pool of their state sponsored health insurance scheme with the central scheme. See table below for the details PFHIS.

**Table:** 

State Name	Name of scheme	Target population	Benefits
Andhra Pradesh	Dr. YSR Arogyashree	BP^1^ Population	Financial coverage of Rs.5 Lakhs per annum for a family
	Arogya Raksha Scheme	AP^2^ Population	Financial coverage of Rs.2 Lakhs per annum for a family. Since 2020 the APL families can choose the insurance coverage amount from a range starting from 1 to 10 lakh based on which the premium has to be paid
Karnataka	Ayushman Baharat Arogya Karnataka Scheme^*^	• BPL households and PDS^3^ cardholders included in the National Food Security Act 2013 • Non- PDS cardholders in APL category	**BPL families-** Cashless treatment through the empanelled hospitals for specified packages, financial assistance up to Rs. 5 Lakhs—per year for a family **APL families**—Coverage on co-payment up to 30% of hospitalization expenses borne by the state government
Kerala	Karunya Arogya Suraksha Paddathi (KASP)- PMJAY	BPL families as per the central and state list	Coverage of Rs.5 Lakhs per year for a family for secondary and tertiary care treatment through empanelled health care providers
Tamil Nadu	Chief Ministers Comprehensive Health Insurance Scheme	Families with income less than Rs.72,000/- per annum	Financial coverage of Rs. 5 Lakhs per family per year
Telangana	Arogyashree Scheme	BPL families	Coverage of INR 1.5 lakhs per year for a family and additional amount of Rs.50 thousand as buffer

^*^The following PFHIS schemes in Karnataka like Vajpayee Arogyashree, Yeshaswini, Rajiv Aogya Bhagya & RSBY has been converged under this scheme to create a single risk pool

^1^BPL – Below Poverty Line is an economic standard which identifies households with lower income, who needs assistance from the government (families with income < 15,000 INR)

^2^APL – Above Poverty Line are those families with annual income more than INR 15,000 but below INR 1 Lakh

^3^PDS – Public Distribution System, food safety program in India

Despite the large coverage of PFHIS in the southern states of India, it is unclear whether coverage is uniform within and across population subgroups. With the announcement of the National Health Protection Scheme in February 2018 by the Indian Government, there appears to be clear policy commitment to an insurance-based model of health financing, even as evidence on the impact of PFHIS is mixed. There are only a handful of studies on national and state level PFHIS on equity dimensions [1, 2, 14, 20, 31], and none of them have applied a gender lens. Given the emphasis being placed on achieving UHC for all in India, we aimed to fill this gap by examining gender-specific socio-economic and educational inequalities in PFHIS coverage in five southern states India namely Andhra Pradesh, Karnataka, Kerala, Tamil Nadu, and Telangana, using fourth and fifth rounds of National Family Health Surveys (NFHS-4, 2015–16, and NFHS-5, 2019–21).

## Data and method

The study used the fourth and fifth rounds of National Family Health Survey (NFHS), which were conducted during 2015–2016 and 2019–2021 respectively. The International Institute for Population Sciences (IIPS), Mumbai, served as the national nodal agency for the NFHS- which was carried out under the supervision of the Ministry of Health and Family Welfare (MoHFW), Government of India, with technical assistance from Inner City Fund (ICF) International United States (US). The NFHS-4 and NFHS-5 samples were designed to provide national, state/union territory, and district-level estimates of various indicators critical to monitor the Sustainable Development Goals (SDGs) on population, health, health insurance nutrition, gender equality, and other development issues. Detailed information on the sampling design of the NFHS-4 and NFHS-5 is available elsewhere [22, 23]. The study used individual recoded files, which covered information of PFHIS from 803,211 individuals (103,525 men, 699,686 women) in NFHS-4 and 817,382 individuals (93,267 men, 724,115 women) aged 15–49 years in NFHS-5. Datasets are not available for gender groups other than “men” and “women,” (eg. trans or other), so our analysis was restricted to these two groups. The study is focused on the five southern states of India, i.e., Andhra Pradesh, Karnataka, Kerala, Tamil Nadu, and Telangana. The final sample was restricted to 97,010 in NFHS-4 and 118,858 in NFHS-5 for the southern states (see Table 1).

**Table 1:** Sample Size for study population aged 15–49 years in Southern states of India

**Southern States**	**NFHS-4, 2015–16**		**NFHS-5, 2019–21**	
	**Male**	**Female**	**Male**	**Female**
Andhra Pradesh	1399	10,428	1,396	10,975
Karnataka	3760	26,291	4,099	30,455
Kerala	1864	11,033	1,294	10,969
Tamil Nadu	4794	28,820	2,993	25,650
Telangana	1054	7,567	3,509	27,518
Total	12,871	84,139	13,291	1,05,567

Source: NFHS-4, 2015–16 and NFHS-5, 2019–21

### Study variables

In NFHS 4 and 5, there are multiple questions across schedules related to health insurance coverage at the individual and household level. The main dependent variable on coverage of PFHIS among women and men, was drawn from the question, “Are you covered by a health scheme or health insurance”? Respondents were further asked to state the type of health scheme or insurance they were covered under. The selection choices were ‘Employees State Insurance Scheme’, ‘Central Government Health Scheme’, ‘State Health Insurance Scheme’, ‘Rashtriya Swasthya Bima Yojana’, ‘Community Health Insurance Programme’, ‘Other Health Insurance through Employer’, ‘Medical Reimbursement from Employer’, and ‘other Privately Purchased Commercial Health Insurance’. Each respondent’s PFHIS status was defined in our study as a binary variable; “covered” if the respondent was covered by any state health insurance scheme (SHIS) or national level health insurance scheme (Rashtriya Swasthya Bima Yojana) for men and women aged 15–49.

### Inequality dimensions

**Fig. 1:**
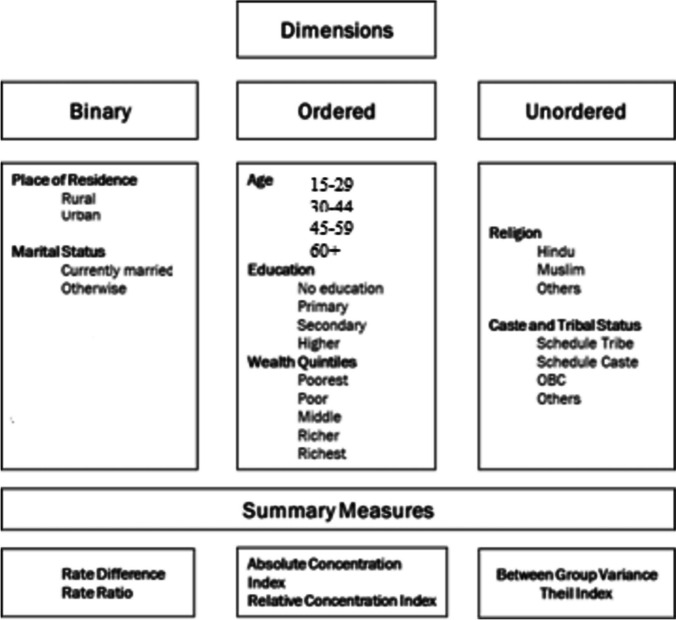
Pictorial representation of the dimensions and summary measures used

The dimensions considered in our study were binary (1), ordered (3) and unordered (3) (see Fig. 1). Binary dimensions were coded as (i) place of residence: urban (reference group) and rural.

*Ordered dimensions were identified* as (i) age: 15–19, 20–24, 25–34, 35–49, (ii) Education: no education, primary, secondary, higher), (iii) Wealth: poorest, poorer, middle, richer, richest. The wealth index was constructed through principal component analysis using information on household assets including the possession of a number of consumer goods and housing characteristics [32, 33].The index was constructed by first dividing asset information into sets of dichotomous variables and indicator weights were assigned using principal component analysis (PCA). The wealth index thus derived was divided into five quintiles: poorest, poorer, middle, richer and richest [34].

*Unordered dimensions* were identified as (i) marital status: currently married (reference group), never married, and others (separated, divorced etc.), (ii) Religion: Hindu (reference group) Muslim, Christian and Others, and (iii) Social Group: Other castes (reference group), Schedule Tribes (ST), Schedule Castes (SC) and Other Backward Classes (OBC).‘Scheduled Tribe’ and ‘Scheduled Caste’ are the tribal and caste groups recognized by the President of India according to article numbers 341 and 342 of the Constitution of India [35]. ‘Backward Class’ is the term used by the Government of India to classify groups that are educationally or socially disadvantaged [36].

### Statistical analysis

Descriptive estimates (i.e., mean, standard errors and 95% confidence intervals) of men and women covered by PFHIS disaggregated by seven dimensions of inequality were obtained for five southern Indian states (Andhra Pradesh (AP), Karnataka, Kerala, Tamil Nadu (TN) and Telangana), using Stata®17 MP version (StataCorp LLC, Lakeway Drive College Station, Texas, USA), using the appropriate sampling weight variables in the dataset due to complex survey design of NFHS surveys. The complex survey design effects were adjusted by using Stata svyset and svy commands. We used two sample Z-test for proportions method to test the significance of change in the PFHIS coverage from NFHS-4 to NFHS-5 [37].

Summary measures of inequality were computed separately for men and women aged 15 + using the World Health Organization’s Health Equity Assessment Toolkit (HEAT) Plus [38, 39]. Absolute and relative summary measures were obtained. Differences and Ratio were used for computing inequalities in binary dimensions: place of residence, and marital status. Absolute Concentration Index (ACI) and Relative Concentration Index (RCI) were used to compute inequalities in the ordered dimensions of age, education and wealth. Between Group Variation (BGV) and Theil Index (TI) were used to compute inequalities in unordered dimensions: religion and caste (see Table 2 for interpretation of these summary measures).

**Table 2:** Summary measures used in the analysis using HEAT tool

Summary Measure	Type	Usage in the analysis	Interpretation
Difference	Absolute-simple	Difference shows the absolute difference between two subgroups. It is used to analyse binary outcomes	A difference of zero shows no inequality. Summary measure greater than zero shows greater concentration of PFHIS coverage among the advantaged groups
Ratio	Relative-Simple-	Ratio shows relative inequality between two subgroups. In this study, it is used to analyse binary outcomes	Ratio more than one show greater magnitude of inequality in comparison to the reference variable. Ratio is one in case of no inequality
Absolute Concentration Index (ACI)	Absolute, Complex and ordered	Absolute concentration index is obtained by calculating the covariance between our health variable (PFHIS) and the individual’s rank in the social economic distributions. Further, this covariance is multiplied by 2 and divided by the mean level of health to get the summary measure	The ACI varies between -100 to 100 and positive values signifies concentration of PFHIS among the richer households. ACI of zero depicts no inequality
Relative concentration index (RCI)	Relative, Complex and ordered	RCI, a complex and relative measure summarises inequality across social economic distributions that can be ordered. It is calculated by dividing the absolute concentration index by mean level of health and multiplied by 100 for better interpretation. The RCI is twice the area between the concentration curve (38)	The RCI varies between + 100 and -100 with zero depicting equal distribution and large positive values indicates higher coverage among the richer households and larger negative values indicating higher coverage among poor households
Between Group Variance (BGV)	Weighted, complex, non-ordered dimension	Between group variance is calculated as the weighted sum of squared difference of the subgroup estimates and the setting average	BGV is always positive with higher values denoting higher inequalities. BGV
Theil Index (TI)	Relative, complex, non-ordered dimension	Theil’s T statistic is population weighted and measures general disproportions. A subgroup’s population share (for eg. subgroup’s population / total weighted States population), the ratio of a state’s average PFHIS coverage among subgroup’s and overall average PFHIS coverage and the natural logarithm of this ratio, are multiplied; and then these products for each dimension are added to get a statistic. Estimates were multiplied by 1000 for better understanding	If there is no inequality, it takes the value zero. Greater absolute values indicate greater levels of inequality

## Results

Table S1- S5 present descriptive characteristics for PFHIS coverage among women and men aged 15–49 years according to their socio-demographic characteristics across Southern states of India during NFHS-4 (2015–16) and NFHS-5 (2019–21). Overall, PFHIS coverage increased significantly among women and men in Andhra Pradesh [Women: 68.11% to 71.05%, *p* < 0.01; Men: 72.89% to 82.88%, *p* < 0.001], and Kerala [Women: 34.60% to 42.03%, *p* < 0.001; Men: 24.56% to 42.37%, *p* < 0.001] from NFHS-4 to NFHS-5. In the case of Karnataka, PFHIS coverage decreased significantly among women [16.42% to 6.01%, *p* < 0.001] and men [22.45% to 6.52%, *p* < 0.001]. In Tami Nadu, PFHIS coverage decreased significantly among women [36.60% to 27.50%, *p* < 0.001] and increased significantly among men [37.03% to 41.97%, *p* < 0.001] from NFHS-4 to NFHS-5. Similarly, in Telangana, PFHIS coverage slightly increased among women [56.82% to 59.85%, *p* < 0.001] from NFHS-4 to NFHS-5, whereas in men, the difference in PFHIS coverage was not significant. The detailed results of the PFHIS coverage among women and men aged 15–49 by background characteristics is presented in Supplementary Tables S1- S5.

### Summary measures of inequality in PFHIS coverage

Table 3 presents the estimates of the summary measures of inequality in PFHIS coverage for binary dimension such as place of residence (Urban, Rural) for men and women. It also presents the changes in the estimates of summary measures of inequality from NFHS-4 to NFHS-5 in both sexes. PFHIS coverage was high in rural populations irrespective of gender across all the states in both surveys. In all states, the magnitude of urban–rural inequality in PFHIS coverage was higher among women in both surveys compared to men. Urban–rural inequality in PFHIS coverage has declined in Andhra Pradesh [Women: -23.08; Men: -21.47 in NFHS-4 to Women: -15.82; Men: -11.52 in NFHS-5] and Telangana [Women: -27.40; Men: -21.81 in NFHS-4 to Women: -23.23; Men: -17.99 in NFHS-5] from NFHS-4 to NFHS-5, whereas, in Kerala and Tamil Nadu, it has increased.The urban–rural ratio of PFHIS coverage was lower among men (Ratio: 0.47) and women (Ratio: 0.54) in Karnataka followed by Telangana (Women: Ratio: 0.61) in NFHS-4 than the others states. In Karnataka, urban–rural difference and urban–rural ratio in PFHIS coverage among women was positive and more than one in NFHS-5, suggesting that PFHIS coverage was concentrated in urban areas among women.

**Table 3:** Summary measures of inequality in PFHIS coverage for binary dimensions between men and women in selected states in NFHS-4, 2015–16 and NFHS-5, 2019–21

	**NFHS-4**				**NFHS-5**				
	**Women**		**Men**		**Women**		**Men**		
	**Difference**	**Ratio**	**Difference**	**Ratio**	**Difference**	**Ratio**	**Difference**		**Ratio**
**Place of residence (Ref: Urban)**									
Andhra Pradesh	-23.08	0.69	-21.47	0.73	-15.82	0.79	-11.52		0.87
Karnataka	-9.46	0.54	-15.70	0.47	0.90	1.16	-4.50		0.46
Kerala	-8.72	0.77	-8.53	0.70	-12.86	0.73	-12.56	0.74	
Tamil Nadu	-6.91	0.83	-5.83	0.85	-9.14	0.71	-4.89		0.85
Telangana	-27.40	0.61	-21.81	0.72	-23.23	0.66	-17.99		0.76

Difference: urban–rural; ratio: urban/rural

Table 4 presents the estimates of the summary measures of inequality in PFHIS coverage for ordered dimensions such as age, education and wealth quintile for men and women in NFHS-4 to NFHS-5. Results showed that PFHIS coverage was greater among older women and men across all states in both surveys. It was observed that age related absolute and relative inequality in PFHIS coverage increased from NFHS-4 to NFHS-5 among both sexes across selected states, however in Kerala, relative inequality decreased from NFHS-4 to NFHS-5 [RCI_NFHS-4_ = 3.41; RCI_NFHS-5_ = 2.47].

**Table 4:** Summary measures of inequality in PFHIS for ordered dimensions between men and women in selected states in NFHS-4, 2015–16 and NFHS-5, 2019–21

	**NFHS-4**				**NFHS-5**			
	**Women**		**Men**		**Women**		**Men**	
	**ACI**	**RCI**	**ACI**	**RCI**	**ACI**	**RCI**	**ACI**	**RCI**
**Age**								
Andhra Pradesh	2.98	4.37	0.31	0.42	4.57	6.44	1.05	1.27
Karnataka	0.49	2.97	0.49	2.17	0.49	8.08	0.14	2.10
Kerala	1.22	3.52	0.84	3.41	1.84	4.37	1.05	2.47
Tamil Nadu	1.34	3.65	1.71	4.62	2.14	7.78	2.74	6.53
Telangana	2.23	3.92	0.12	0.17	3.56	5.95	0.27	0.41
**Education**								
Andhra Pradesh	-5.48	-8.05	-3.21	-4.40	-5.57	-7.84	-2.58	-3.12
Karnataka	-1.28	-7.82	-1.40	-6.22	0.11	1.77	-0.51	-7.78
Kerala	-2.49	-7.21	-2.34	-9.54	-2.90	-6.89	-1.99	-4.69
Tamil Nadu	-1.79	-4.90	-1.18	-3.20	-2.35	-8.55	-1.70	-4.05
Telangana	-6.95	-12.23	-3.74	-5.58	-5.97	-9.98	-1.55	-2.30
**Wealth quintile**								
Andhra Pradesh	-5.64	-8.28	-4.94	-6.77	-3.42	-4.81	-1.87	-2.26
Karnataka	-2.73	-16.61	-3.22	-14.33	0.45	7.52	-1.41	-21.71
Kerala	-5.46	-15.78	-5.01	-20.42	-6.03	-14.36	-5.87	-13.84
Tamil Nadu	-1.34	-3.67	-0.94	-2.55	-2.27	-8.26	-1.85	-4.41
Telangana	-7.64	-13.46	-5.23	-7.79	-5.40	-9.02	-2.55	-3.79

In both surveys, the magnitude of absolute and relative age-related inequality was higher among women in Andhra Pradesh, Karnataka, Kerala, and Telangana than men, whereas in Tamil Nadu, this magnitude was higher among men [ACI_NFHS-4_ = 1.71; ACI_NFHS-5_ = 2.74].

Education-related inequality had a negative gradient, meaning higher PFHIS coverage among less educated women and men in both surveys. Education related absolute inequality has increased among women from NFHS-4 to NFHS-5 in Andhra Pradesh, Kerala, and Tamil Nadu, whereas relative inequality has decreased in Andhra Pradesh, Kerala, and Telangana from NFHS-4 to NFHS-5. In case of men, absolute and relative inequality decreased in Andhra Pradehsh, Kerala, and Telangana from NFHS-4 to NFHS-5. Education-related absolute and relative inequality in PFHIS coverage was higher among less educated women as compared to men in NFHS-4 and NFHS-5 in Andhra Pradesh, Tamil Nadu, and Telangana. In case of Karnataka, ACI and RCI was concentrated among higher educated women in NFHS-5, whereas in NFHS-4 ACI and RCI was concentrated among less educated women. The magnitude of education-related relative inequality was twice as high as among women in Telangana (RCI_NFHS-4_: -12.23; RCI_NFHS-5:_ -9.98) and Andhra Pradesh (RCI_NFHS-4_: -8.05; RCI_NFHS-5:_ -7.84) as compared to men in Telangana (RCI_NFHS-4_: -5.58; RCI_NFHS-5:_ -2.30) and Andhra Pradesh (RCI_NFHS-4_: -4.40; RCI_NFHS-5:_ -3.12) and this inequality remained same in NFHS-5. In Kerala, there was a reverse scenario where the level of education-related relative inequality was higher among men (RCI_NFHS-4_: -9.54) in comparison to women (RCI_NFHS-4_: -7.21) in NFHS-4, whereas, in NFHS-5, education-related relative inequality was higher among women (RCI_NFHS-5_: -6.89) in comparison to men (RCI_NFHS-5_: -4.69).

Wealth related inequality also had a negative gradient (in favour of the poor), but the level of inequality was higher among women across all selected states except Karnataka using absolute and relative measures in NFHS-5. It was observed that in Andhra Pradesh and Telangana, magnitude of wealth related absolute and relative inequality has decreased from NFHS-4 to NFHS-5. In Tamil Nadu, the value of ACI and RCI increased from NFHS-4 to NFHS-5 among both women and men and the inequality was higher among women as compared to men in both survey, even as the magnitude of inequality and gender differences was lower than that observed in other southern states. High magnitude of wealth related relative inequality was observed in Kerala women (RCI_NFHS-4_: -15.78; RCI_NFHS-5_: -14.36) and men (RCI_NFHS-4_: -20.42; RCI_NFHS-5_: -13.84), whereas this inequality has decreased from NFHS-4 to NFHS-5. In Karnataka, the situation is slightly different than the others southern states, as the value of ACI and RCI in NFHS-4 suggest that PFHIS coverage was concentrated in poor women, whereas in NFHS-5, coverage was concentrated in rich women. Similarly, in case of men in Karnataka, the magnitude of ACI decreased, and RCI increased from NFHS-4 to NFHS-5.

Table 5 presents the estimates of the summary measures of inequality in PFHIS coverage for unordered dimensions such as marital status, caste and religion for women and men in NFHS-4 and NFHS-5. Inequality related to marital status was very small or not present among men and women in all selected states across both rounds of the survey. PFHIS coverage was higher among socially disadvantaged groups across all states except Karnataka among both sexes in both surveys (Supplementary tables S1- S5). Caste-based absolute inequalities were most pronounced in Telangana and Kerala among women and men in both surveys. In Kerala, caste-based absolute inequalities were of greater magnitude among women (BGV _NFHS-4_ = 45.42; BGV _NFHS-5_ = 36.69) as compared to men (BGV _NFHS-4_ = 28.47; BGV _NFHS-45_ = 16.80) in both surveys. In Telangana, caste-based absolute inequalities in PFHIS coverage were higher among women (BGV _NFHS-4_ = 72.26) compared to men (BGV _NFHS-4_ = 69.13) in NFHS-4, whereas in NFHS-5, absolute inequalities were higher among men (BGV_NFHS-5_ = 43.44) compared to women (BGV_NFHS-5_ = 27.20), however, the magnitude of inequalities declined from NFHS-4 to NFHS-5. Religion-related absolute inequalities in PFHIS coverage were higher in Kerala with greater magnitude in women (BGV_NFHS-4_ = 49.91) than men (BGV _NFHS-4_ = 17.47) in NFHS-4, whereas, in NFHS-5, these inequalities increased in women (BGV_NFHS-5_ = 51.30) and men (BGV_NFHS-5_ = 17.65). These inequalities were also present in women (BGV _NFHS-4_ = 18.05; BGV_NFHS-5_ = 15.61) and men (BGV _NFHS-4_ = 17.26; BGV_NFHS-5_ = 13.42) belonging to Telangana in both surveys.

**Table 5:** Summary measures of inequality in PFHIS for unordered dimensions between men and women in selected states in NFHS-4, 2015–16 and NFHS-5, 2019–21

	**NFHS-4**				**NFHS-5**			
	**Women**		**Men**		**Women**		**Men**	
	**BGV**	**Theil Index**	**BGV**	**Theil Index**	**BGV**	**Theil Index**	**BGV**	**Theil Index**
**Marital Status**								
Andhra Pradesh	8.48	0.90	0.05	0.01	5.12	0.49	0.58	0.04
Karnataka	0.19	0.35	4.57	5.33	0.09	1.26	0.01	0.07
Kerala	5.04	1.97	4.50	3.75	1.00	0.28	3.10	0.83
Tamil Nadu	1.42	0.52	7.43	2.78	1.79	1.13	19.69	5.55
Telangana	14.49	2.19	3.14	0.35	14.56	1.97	2.62	0.29
**Social Group**								
Andhra Pradesh	15.78	1.70	7.82	0.73	8.90	0.89	1.93	0.14
Karnataka	1.57	2.84	4.86	4.06	0.92	11.03	1.83	18.04
Kerala	45.42	16.97	28.47	22.76	36.69	9.87	16.80	4.40
Tamil Nadu	10.92	4.01	6.40	2.52	4.32	2.83	19.43	6.50
Telangana	72.26	11.94	69.13	7.74	27.20	4.05	43.44	5.14
**Religion**								
Andhra Pradesh	6.04	0.65	10.01	0.92	0.68	0.07	7.97	0.59
Karnataka	1.01	2.10	2.17	2.45	0.61	9.53	0.31	4.05
Kerala	49.91	21.45	17.47	15.40	51.30	14.84	17.65	4.97
Tamil Nadu	1.61	0.63	17.84	7.57	4.93	3.56	12.08	4.00
Telangana	18.05	3.00	17.26	2.21	15.61	2.31	13.42	1.53

## Discussion

The study explored the gender-specific inequalities in PFHIS coverage in southern states of India as they intersected with other axes of inequality like place of residence, marital status, age, education, wealth status, religion, and caste. As a reference point or benchmark was absent to compare the inequalities, magnitudes of inequality were compared across states using absolute and relative summary measures.

Overall, men had higher level of PFHIS coverage than women especially in Andhra Pradesh, Tamil Nadu, and Telangana. However, within group inequalities were of greater magnitude among women. The pattern of inequality by place of residence was also not different by sex (using both absolute and relative measures): rural men and women reported more PFHIS coverage than their urban counterparts across all selected states in both surveys. In both surveys, the urban–rural inequality in PFHIS coverage was more concentrated in rural women as compared to rural men. The urban–rural inequality in PFHIS coverage decreased from NFHS-4 to NFHS-5 in Andhra Pradesh, and Telangana in both sexes suggestive of increasing coverage in urban areas, whereas, in Kerala and Tamil Nadu, it has increased, suggestive of growing rurality of coverage.

We found that PFHIS coverage was concentrated in older women and men in both surveys across all states. Age-related inequality was higher among women compared to men in both surveys in Andhra Pradesh, Karnataka, Kerala, and Telangana and these inequalities increased from NFHS-4 to NFHS-5.

Education related relative concentration of PHFIS coverage was higher among less educated women and men in NFHS-5 across all states except Karnataka. Education-related absolute and relative inequality in PHFIS were not significantly different among women as compared to men across all states in NFHS-4, whereas, in NFHS-5, this magnitude of inequality was higher among women than their men counterparts. In Kerala, education-related relative inequality was higher among men compared to women in NFHS-4, whereas, in NFHS-5, the situation changed, and this relative inequality became higher in women compared to men. These findings may need to be interpreted with caution as in this state, the sample size for no schooling was small, given the state’s high literacy rate.

Wealth related absolute and relative inequality in PFHIS coverage were higher with coverage being mainly concentrated in poor women and men population across all selected states in both surveys. However, the level of inequality was higher among women compared to men except Karnataka. Findings revealed that the magnitude of wealth related relative inequality was higher in men compared to women in NFHS-4 in Kerala, whereas in NFHS-5, the wealth related relative inequality was slightly higher in women than men, and the magnitudes of these wealth inequalities declined from NFHS-4 to NFHS-5. This shows us that the PFHIS coverage is skewed towards the poor across sexes (more so among poor women). Here, too, given that wealth was calculated in quintiles across all four states, the result was that persons belonging to poor/poorest category was very low in this state. So while results should be interpreted with caution, findings do suggest that PFHIS coverage is pro-poor, which is what one would expect given their design. A number of state specific strategies were adopted to improve the coverage under PFHIS during the rollout of RSBY. At the national level insurance providers were entrusted with responsibility of generating awareness about the scheme and to engage in Information, Education and Communication activities during the time and after the enrolment [40]. Studies from Kerala found that local self-governments (grama panchayats) acted as the main source for receiving information on RSBY-CHIS in the state and their involvement, along with the support of Kudumbasree (women self-help group network) facilitated improved enrolment of households under the scheme [41, 42]. While in Andhra Pradesh, Arogya Mithras were appointed as patient advocates, to support the patient to access quality healthcare and for community outreach [43]. The state of Karnataka introduced outreach via health camps for improving health and financial wellbeing of the target population [44]. We also found that in Tamil Nadu, the coverage of PFHIS among women was dcreasing while the coverage of men has increased. Contrary to this the current status in the PMJAY dashboard shows higher coverage among women (52%) than men (47%) in Tamil Nadu.( https://dashboard.pmjay.gov.in/publicdashboard/#/) So the trend in coverage is varying across years there are intiavties are underway like “Ayushman bhav” aiming to cover the leftout population. (https://ayushmanbhav.mohfw.gov.in/) Further state specific studies can be undertaken to understand the gender based coverage in enrolment under PFHIS.

Caste based inequality was higher among women belonging to socially disadvantaged groups compared to men belonging to socially disadvantaged groups in both surveys, however, the magnitude of inequality decreased from NFHS-4 to NFHS-5 except Karnataka. Findings revealed that magnitude of caste based absolute inequalities declined from NFHS-4 to NFHS-5 among both sexes in Kerala and Telangana, whereas inequalities was higher than the other states. This may be due to higher coverage among socially disadvantaged groups and lower coverage among others. Religion related absolute inequality in PFHIS coverage was higher in Kerala with greater magnitude in women than men and these inequalities has increased from NFHS-4 to NFHS-5. Religion related inequality in PFHIS coverage was higher in Kerala and Telangana among women compared to men in both surveys whereas, these inequalities increased in PFHIS coverage increased from NFHS-4 to NFHS-5. This may be due to the fact that coverage among women and men has increased in each religion, the highest increment in coverage was observed among women who belong to Other religious group, similarly Hindu and Other religious group of men were covered more than the Muslim religion.

In Supplementary table S6, we present a table summarizing the change in inequality over time. Broadly speaking, we found that the magnitudes of inequality in binary variables (place of residence and marital status) were likely too small to be of public health significance and further that rural coverage was favoured, which is mostly desirable, except in situations where there are large urban poor populations (which become invisible in aggregate) [45–47].We further found that there were increases in inequality in our ordinal dimensions (age, education and wealth) in three of our four states, but that this increase reflected greater coverage among less educated, poorer and older populations, which is to a large extent desirable. This pattern was seen more among women than men. Finally, and most concerningly, we saw large magnitudes of inequality by social group and religion, increasing in both absolute and relative terms for both men and women. The only groups for which social group and religion-based inequality decreased was women in Telangana and men in Kerala. This last pattern in particular is worrisome and definitely warrants further study.

A national analysis of NFHS-3 survey found out that coverage (at least one household member covered by health insurance) was higher among urban households, better educated individuals and those in the wealthiest quintile [48]. Our findings from the year 2015 and 2019 showed that coverage of PFHIS had managed to reach socially and economically deprived groups of the society over a period of time. PFHIS coverage in our study mostly favoured socioeconomically deprived communities, the uneducated, poor, and elderly. A study assessing equity in Ayushman Bharat found out that the possession of an active RSBY card has promoted equity within the household and across social groups [49]. However, our study revealed nuanced state level patterns: for instance, coverage in Kerala was higher in women than men, yet within group inequalities persisted. Place of residence related inequality measured indicated greater coverage in rural areas among all the select states. All PFHIS such as RSBY are designed to increase coverage among families from rural areas living below poverty line and have also resulted in major increase in insurance coverage of the population [50]. The study also found very small inequalities by marital status in Tamil Nadu and Kerala. Married women were underinsured even when compared to never married men in all states except Kerala. It is usually said that women compromise their own health needs in most instances by prioritizing the health of main bread-winners of the household-usually the men members of the household) [1]. However, the situation of women outside of marriage appears to be worse from a PFHIS coverage perspective. A secondary data study examining Rajiv Aarogyasri Health Insurance Scheme in AP in 2014 reported that beneficiaries who were either illiterate or had a rural address utilised the scheme mostly and provided more protection to the poor people [43]. Our findings show that PFHIS coverage was concentrated among less educated but inequalities were visible in all selected states and more among women than men.

This study tried to provide evidence to examine PFHIS coverage from a gender perspective. We have provided mainly gender-disaggregated descriptive data using established categories from an existing data set within age group of 15–49. We were unable, therefore, to speak to the processes of exclusion that relate to the inequalities and explain inequities using a broader perspective [51]. This study was based on self –reported data, which could also under or misreport coverage. Data was not collected for transgender persons. Hence, addressing the health equities among genders other than men and women was not possible. NFHS has a disclaimer that readers should be cautious while interpreting and comparing the trends as some states may have smaller sample sizes especially in case of men, where sample of men is very small. Moreover, NFHS-5 does not fully capture the transformative interventions of Ayushman Bharat -Pradhan Mantri Jan Aarogya Yojana and Pradhan Mantri-Surakshit Matritva Abhiyan as they were being rolled out as households were being surveyed across the country.

Women’s health risks range from their reproductive roles to vulnerabilities due to infectious, chronic and non-communicable diseases that can disproportionately affect them because of their greater longevity [3]. Many of the problems women faced when utilising the benefits of RSBY were related to the design of RSBY where healthcare needs [52] of women were compromised due to the cap. Ayushman Bharat, India’s redesigned PM-JAY, aims to provide an annual health cover of up to Rs. 5 lakh to vulnerable 10 crore households identified as beneficiaries using the Socio Economic and Caste Census database [53]. It is assumed that RSBY and other PFHIS will provide coverage to the entire household in a gender-neutral and unbiased fashion. Proceeding with this, this scheme will require addressing the fact that, as seen from NFHS-4 and NFHS-5 data, it is not only women from poor households but also educated and women from middle- and upper-income households who encounter financial barriers [22, 23]. Moreover, various minoritized groups like caste and religious minorities as well as tribal persons may be facing disadvantage in coverage across southern Indian states – this is an area requiring greater study. In fact, our study did not look at these intersectionality due to sample size restrictions, but our findings suggest that it will be very important to carry out well-powered state-specific studies on PFHIS coverage on the basis of caste, indigeneity, and religion.

Looking at healthcare through a gender lens, PFHIS should not only focus on whether insurance should be equally provided to men and women but on how to equitably meet individual needs of population subgroups by gender, and ideally gender. We need to put additional efforts to make the scheme more accessible to women by focusing on reducing the gender inequalities that exist in caste, religion, education etc. among these better performing states of India. Furthermore, there is a lack of data and comprehensive research on how these financial protection programmes benefit women. Therefore, impact evaluations on the range of these programmes for women and their impact on other financial protection indicators (like service utilisation) and population health outcomes (like mortality) need to be conducted.

## Conclusion

We found inequalities in self-reported men and women enrolment in five southern states with robust public insurance schemes. Inequalities favour the poor, uneducated, unemployed and elderly, which is desirable. However, religion, caste-based, age and education-based inequalities exist across genders, and are at times greater among women, which demands further study. Pro-poor inequalities suggest that horizontal equity is being enhanced in PHFIS, and yet there is a way to go for these schemes to truly offer financial risk protection by addressing the intersecting marginalizations faced by persons across gender categories.

### Supplementary Information

**Additional file 1:Table S1: **Percentage distribution of women and men covered by PFHIS according to background characteristics in Andhra Pradesh in NFHS-4 and NFHS-5. **Table S2.** Percentage distribution of women and men covered by PFHIS according to background characteristics in Karnataka in NFHS-4 and NFHS-5. **Table S3.**  Percentage distribution of women and men covered by PFHIS according to background characteristics in Kerala in NFHS-4 and NFHS-5. **Table S4.**  Percentage distribution of women and men covered by PFHIS according to background characteristics in Tamil Nadu in NFHS-4 and NFHS-5. **Table S5.**  Percentage distribution of women and men covered by PFHIS according to background characteristics in Telangana in NFHS-4 and NFHS-5. **Table S6.** Inequality change between NFHS4 and NFHS 5.

## Data Availability

All data used in the study is archived in the public repository of Demographic and Health Survey (DHS). The data can be accessed using: https://dhsprogram.com/data/dataset_admin/index.cfm, which requires registration.

## Abbreviations

NCDs: Non-Communicable Diseases
ACI: Absolute Concentration Index
BGV: Between Group Variance
TI: Theil Index
RCI: Relative concentration index
PFHIS: Publicly funded health insurance scheme
