# Supplementary material for: Gender-specific inequalities in coverage of Publicly Funded Health Insurance Schemes in Southern States of India: evidence from National Family Health Surveys
Source: BMC Public Health. 2023 Dec 4;23:2414. doi: 10.1186/s12889-023-17231-0 (PMC10696875; doi:10.1186/s12889-023-17231-0)
Supplement: Supplementary file 1 — Additional file 1:Table S1: Percentage distribution of women and men covered by PFHIS according to background characteristics in Andhra Pradesh in NFHS-4 and NFHS-5. Table S2. Percentage distribution of women and men covered by PFHIS according to background characteristics in Karnataka in NFHS-4 and NFHS-5. Table S3. Percentage distribution of women and men covered by PFHIS according to background characteristics in Kerala in NFHS-4 and NFHS-5. Table S4. Percentage distribution of women and men covered by PFHIS according to background characteristics in Tamil Nadu in NFHS-4 and NFHS-5. Table S5. Percentage distribution of women and men covered by PFHIS according to background characteristics in Telangana in NFHS-4 and NFHS-5. Table S6. Inequality change between NFHS4 and NFHS 5. [file 12889_2023_17231_MOESM1_ESM.docx]

**Supplementary**

| **Table S1: Percentage distribution of women and men covered by PFHIS according to background characteristics in Andhra Pradesh in NFHS-4 and NFHS-5** | | | | | | | | | | |
| --- | --- | --- | --- | --- | --- | --- | --- | --- | --- | --- |
|  | **Women** | | | | | **Men** | | | | |
|  | **NFHS-4** | | **NFHS-5** | |  | **NFHS-4** | | **NFHS-5** | |  |
| **Background characteristics** | **N** | **%** | **N** | **%** | **p value** | **N** | **%** | **N** | **%** | **p value** |
| **Place of Residence** |  |  |  |  |  |  |  |  |  |  |
| Urban | 3298 | 52.33 | 3468 | 60.23 | p<0.001 | 488 | 58.91 | 441 | 75.01 | p<0.001 |
| Rural | 7130 | 75.41 | 7507 | 76.05 | p>0.100 | 911 | 80.37 | 955 | 86.52 | p<0.001 |
| **Age** |  |  |  |  |  |  |  |  |  |  |
| 15-19 | 1329 | 69.89 | 1362 | 63.71 | p<0.001 | 201 | 76.15 | 228 | 82.14 | p>0.100 |
| 20-24 | 1742 | 55.14 | 1536 | 57.03 | p>0.100 | 200 | 76.45 | 178 | 82.51 | p>0.100 |
| 25-34 | 3173 | 64.58 | 3409 | 67.01 | p<0.050 | 416 | 65.50 | 430 | 79.13 | p<0.001 |
| 35-49 | 4183 | 75.63 | 4668 | 80.76 | p<0.001 | 582 | 75.83 | 560 | 86.19 | p<0.001 |
| **Education** |  |  |  |  |  |  |  |  |  |  |
| No schooling | 3485 | 79.76 | 3056 | 84.08 | p<0.001 | 238 | 78.23 | 247 | 89.74 | p<0.001 |
| Primary | 1488 | 72.99 | 1503 | 79.12 | p<0.001 | 178 | 80.95 | 187 | 85.87 | p>0.100 |
| Secondary | 4282 | 61.99 | 4938 | 65.44 | p<0.001 | 701 | 73.93 | 700 | 83.09 | p<0.001 |
| Higher | 1173 | 49.65 | 1478 | 54.67 | P<0.010 | 282 | 60.70 | 262 | 73.76 | p>0.100 |
| **Wealth** |  |  |  |  |  |  |  |  |  |  |
| Poorest | 384 | 75.17 | 450 | 73.51 | p>0.100 | 45 | 78.12 | 55 | 84.74 | p>0.100 |
| Poorer | 1477 | 76.98 | 1996 | 78.01 | p>0.100 | 171 | 84.29 | 226 | 81.99 | p>0.100 |
| Middle | 3442 | 76.45 | 3424 | 74.48 | p>0.100 | 439 | 78.97 | 453 | 86.64 | p<0.001 |
| Richer | 3279 | 68.30 | 3344 | 71.66 | p<0.010 | 467 | 73.66 | 435 | 87.06 | p<0.001 |
| Richest | 1845 | 43.65 | 1762 | 54.73 | p<0.001 | 277 | 54.05 | 228 | 67.89 | p<0.001 |
| **Caste** |  |  |  |  |  |  |  |  |  |  |
| Schedule tribe | 596 | 73.84 | 464 | 74.74 | p>0.100 | 94 | 78.15 | 59 | 80.04 | p>0.100 |
| Schedule caste | 2122 | 73.61 | 2472 | 73.61 | p>0.100 | 243 | 77.49 | 273 | 81.00 | p>0.100 |
| Other Backward Class | 5389 | 67.87 | 5603 | 71.99 | p<0.001 | 724 | 71.87 | 785 | 84.05 | p<0.001 |
| Other | 2319 | 62.22 | 2432 | 65.68 | p<0.010 | 331 | 70.19 | 279 | 82.06 | p<0.001 |
| **Religion** |  |  |  |  |  |  |  |  |  |  |
| Hindu | 8738 | 68.15 | 9051 | 70.68 | p<0.001 | 1193 | 71.57 | 1164 | 83.08 | p<0.001 |
| Muslim | 856 | 61.91 | 810 | 73.33 | p<0.001 | 134 | 80.31 | 111 | 89.04 | p>0.100 |
| Others | 834 | 74.12 | 1114 | 72.41 | p>0.100 | 72 | 80.83 | 121 | 75.35 | p>0.100 |
| **Marital Status** |  |  |  |  |  |  |  |  |  |  |
| Never married | 1616 | 7313 | 1789 | 68.85 | p<0.001 | 454 | 72.75 | 492 | 82.51 | p<0.001 |
| Married | 8044 | 66.53 | 8306 | 70.75 | p<0.001 | 928 | 72.99 | 883 | 82.95 | p<0.001 |
| Other | 768 | 74.11 | 880 | 78.34 | p>0.100 | 17 | 71.05 | 21 | 88.82 | p<0.001 |
| Total | 10428 | 68.11 | 10975 | 71.05 | p<0.001 | 1399 | 72.89 | 1396 | 82.88 | p<0.001 |

| **Table S2: Percentage distribution of women and men covered by PFHIS according to background characteristics in Karnataka in NFHS-4 and NFHS-5** | | | | | | | | | | | |
| --- | --- | --- | --- | --- | --- | --- | --- | --- | --- | --- | --- |
|  | **Women** | | | | | **Men** | | | | | |
|  | **NFHS-4** | | **NFHS-5** | |  | **NFHS-4** | | | **NFHS-5** | |  |
| **Background characteristics** | **N** | **%** | **N** | **%** | **p value** | **N** | **%** | | **N** | **%** | **p value** |
| **Place of Residence** |  |  |  |  |  |  |  | |  |  |  |
| Urban | 11456 | 11.08 | 12361 | 6.55 | p<0.001 | 1688 | 13.80 | | 1709 | 3.89 | p<0.001 |
| Rural | 14835 | 20.54 | 18094 | 5.65 | p<0.001 | 2072 | 29.50 | | 2390 | 8.39 | p<0.001 |
| **Age** |  |  |  |  |  |  |  | |  |  |  |
| 15-19 | 3715 | 16.27 | 4374 | 4.68 | p<0.001 | 589 | 19.40 | | 622 | 5.04 | p<0.001 |
| 20-24 | 4347 | 14.76 | 4365 | 4.77 | p<0.001 | 597 | 22.19 | | 579 | 7.70 | p<0.001 |
| 25-34 | 8630 | 15.84 | 9539 | 6.02 | p<0.001 | 1137 | 23.70 | | 1183 | 6.48 | p<0.001 |
| 35-49 | 9599 | 17.75 | 12177 | 6.93 | p<0.001 | 1437 | 22.82 | | 1716 | 6.68 | p<0.001 |
| **Education** |  |  |  |  |  |  |  | |  |  |  |
| No schooling | 6268 | 18.60 | 5904 | 5.24 | p<0.001 | 430 | 25.40 | | 432 | 8.40 | p<0.001 |
| Primary | 2741 | 19.31 | 2850 | 6.37 | p<0.001 | 447 | 22.95 | | 396 | 9.37 | p<0.001 |
| Secondary | 13698 | 16.57 | 17035 | 6.25 | p<0.001 | 2210 | 24.08 | | 2468 | 6.02 | p<0.001 |
| Higher | 3584 | 9.82 | 4666 | 5.89 | p<0.001 | 674 | 14.89 | | 803 | 5.64 | p<0.001 |
| **Wealth** |  |  |  |  |  |  |  | |  |  |  |
| Poorest | 1429 | 18.37 | 1987 | 4.49 | p<0.001 | 182 | 19.90 | | 263 | 9.67 | p<0.001 |
| Poorer | 4931 | 20.70 | 5365 | 5.05 | p<0.001 | 739 | 28.46 | | 678 | 10.27 | p<0.001 |
| Middle | 7174 | 21.44 | 8825 | 5.98 | p<0.001 | 992 | 29.54 | | 1203 | 7.62 | p<0.001 |
| Richer | 7297 | 15.84 | 8422 | 5.88 | p<0.001 | 1102 | 20.30 | | 1182 | 4.88 | p<0.001 |
| Richest | 5459 | 6.21 | 5856 | 7.64 | p<0.010 | 745 | 10.85 | | 773 | 2.93 | p<0.001 |
| **Caste** |  |  |  |  |  |  |  | |  |  |  |
| Schedule tribe | 2692 | 15.89 | 3199 | 6.10 | p<0.001 | 312 | 28.18 | | 366 | 7.97 | p<0.001 |
| Schedule caste | 5601 | 16.32 | 6464 | 6.32 | p<0.001 | 841 | 21.37 | | 789 | 5.82 | p<0.001 |
| Other Backward Class | 12672 | 17.85 | 17236 | 5.60 | p<0.001 | 1723 | 25.91 | | 2328 | 6.23 | p<0.001 |
| Other | 2789 | 14.14 | 2437 | 9.13 | p<0.001 | 497 | 23.48 | | 350 | 10.59 | p<0.001 |
| **Religion** |  |  |  |  |  |  |  | |  |  |  |
| Hindu | 21851 | 16.45 | 26192 | 6.32 | p<0.001 | 3083 | 22.86 | | 3606 | 6.71 | p<0.001 |
| Muslim | 3689 | 17.39 | 3575 | 4.30 | p<0.001 | 563 | 21.87 | | 420 | 5.28 | p<0.001 |
| Others | 751 | 10.86 | 687 | 3.13 | p<0.001 | 113 | 14.33 | | 73 | 3.89 | p<0.050 |
| **Marital Status** |  |  |  |  |  |  |  | |  |  |  |
| Never married | 5525 | 16.02 | 6635 | 5.45 | p<0.001 | 1562 | 21.03 | | 1719 | 6.51 | p<0.001 |
| Married | 19143 | 16.40 | 21891 | 6.15 | p<0.001 | 2144 | 23.83 | 2346 | | 6.51 | p<0.001 |
| Others | 1623 | 18.02 | 1929 | 6.34 | p<0.001 | 54 | 8.86 | | 34 | 7.40 | p<0.001 |
| Total | 26291 | 16.42 | 30455 | 6.01 | p<0.001 | 3760 | 22.45 | | 4099 | 6.52 | p<0.001 |

| **Table S3: Percentage distribution of women and men covered by PFHIS according to background characteristics in Kerala in NFHS-4 and NFHS-5** | | | | | | | | | | | |
| --- | --- | --- | --- | --- | --- | --- | --- | --- | --- | --- | --- |
|  | **Women** | | | | | **Men** | | | | | |
|  | **NFHS-4** | | **NFHS-5** | |  | **NFHS-4** | | **NFHS-5** | | |  |
| **Background characteristics** | **N** | **%** | **N** | **%** | **p value** | **N** | **%** | **N** | | **%** | **p value** |
| **Place of Residence** |  |  |  |  |  |  |  |  | |  |  |
| Urban | 5172 | 29.96 | 5277 | 35.36 | p<0.001 | 872 | 20.02 | 597 | | 35.60 | p<0.001 |
| Rural | 5861 | 38.69 | 5692 | 48.22 | p<0.001 | 992 | 28.55 | 697 | | 48.17 | p<0.001 |
| **Age** |  |  |  |  |  |  |  |  | |  |  |
| 15-19 | 1504 | 31.52 | 1472 | 38.59 | p<0.001 | 307 | 23.09 | 214 | | 36.96 | p<0.001 |
| 20-24 | 1519 | 32.69 | 1426 | 39.70 | p<0.001 | 279 | 23.60 | 181 | | 40.41 | p<0.001 |
| 25-34 | 3171 | 33.08 | 2873 | 37.61 | p<0.001 | 515 | 22.80 | 308 | | 45.27 | p<0.001 |
| 35-49 | 4840 | 37.15 | 5198 | 46.09 | p<0.001 | 763 | 26.69 | 592 | | 43.42 | p<0.001 |
| **Education** |  |  |  |  |  |  |  |  | |  |  |
| No schooling | 101 | 41.95 | 77 | 49.36 | p>0.100 | 11 | 43.41 | 29 | | 50.11 | p>0.100 |
| Primary | 410 | 40.92 | 348 | 52.83 | p<0.001 | 82 | 34.22 | 38 | | 63.68 | p<0.001 |
| Secondary | 7256 | 37.78 | 6922 | 45.75 | p<0.001 | 1267 | 26.78 | 881 | | 43.39 | p<0.001 |
| Higher | 3266 | 26.52 | 3623 | 33.73 | p<0.001 | 504 | 16.97 | 346 | | 36.80 | p<0.001 |
| **Wealth** |  |  |  |  |  |  |  |  | |  |  |
| Poorest | 30 | 31.00 | 71 | 41.99 | p>0.100 | 8 | 0.00 | 11 | | 32.86 | p>0.100 |
| Poorer | 215 | 47.05 | 474 | 55.77 | p>0.100 | 38 | 31.21 | 60 | | 63.62 | p<0.001 |
| Middle | 1396 | 48.39 | 1890 | 57.11 | p<0.001 | 299 | 37.96 | 237 | | 53.67 | p<0.001 |
| Richer | 4069 | 43.40 | 4094 | 47.82 | p<0.001 | 605 | 32.80 | 502 | | 48.12 | p<0.001 |
| Richest | 5323 | 23.77 | 4440 | 28.81 | p<0.001 | 914 | 14.66 | 483 | | 28.43 | p<0.001 |
| **Caste** |  |  |  |  |  |  |  |  | |  |  |
| Schedule tribe | 146 | 57.64 | 176 | 60.76 | p>0.100 | 37 | 34.98 | 30 | | 58.39 | p>0.100 |
| Schedule caste | 1083 | 51.52 | 1211 | 56.20 | p>0.100 | 173 | 33.60 | 138 | | 50.75 | p<0.001 |
| Other Backward Class | 6153 | 34.15 | 6419 | 41.93 | p<0.001 | 919 | 27.85 | 768 | | 40.81 | p<0.001 |
| Other | 3200 | 29.23 | 2734 | 36.18 | p<0.001 | 600 | 18.49 | 308 | | 40.05 | p<0.001 |
| **Religion** |  |  |  |  | p<0.001 |  |  |  | |  |  |
| Hindu | 6229 | 40.76 | 5958 | 48.47 | p<0.001 | 1077 | 27.50 | 739 | | 46.01 | p<0.001 |
| Muslim | 3077 | 25.68 | 3272 | 32.84 | p<0.001 | 520 | 17.91 | 335 | | 37.61 | p<0.001 |
| Others | 1727 | 28.25 | 1739 | 37.28 | p<0.001 | 267 | 25.60 | 220 | | 37.39 | p<0.001 |
| **Marital Status** |  |  |  |  |  |  |  |  | |  |  |
| Never married | 2503 | 32.44 | 2365 | 40.58 | p<0.001 | 854 | 22.25 | 602 | | 40.88 | p<0.001 |
| Married | 8147 | 34.76 | 8163 | 42.26 | p<0.001 | 997 | 26.48 | | 677 | 43.44 | p<0.001 |
| Other | 383 | 45.28 | 441 | 45.57 | p<0.001 | 13 | 27.83 | 15 | | 53.64 | p>0.100 |
| Total | 11033 | 34.60 | 10969 | 42.03 | p<0.001 | 1864 | 24.56 | 1294 | | 42.37 | p<0.001 |

|  | |  | |  | | |  |  | | |  |  |  | | |  |  | | |  |
| --- | --- | --- | --- | --- | --- | --- | --- | --- | --- | --- | --- | --- | --- | --- | --- | --- | --- | --- | --- | --- |
| **Table S4: Percentage distribution of women and men covered by PFHIS according to background characteristics in Tamil Nadu in NFHS-4 and NFHS-5** | | | | | | | | | | | | | | | | | | | | |
|  | | **Women** | | | | | | | | | | **Men** | | | | | | | | |
|  | | **NFHS-4** | | | | | **NFHS-5** | | | |  | **NFHS-4** | | | | **NFHS-5** | | | |  |
| **Background characteristics** | | | **N** | | | **%** | **N** | | | **%** | **p value** | **N** | | | **%** | **N** | | | **%** | **p value** |
| **Place of Residence** | | |  | | |  |  | | |  |  |  | | |  |  | | |  |  |
| Urban | | | 14665 | | | 33.21 | 12361 | | | 22.76 | p<0.001 | 2511 | | | 34.26 | 1432 | | | 37.58 | p<0.050 |
| Rural | | | 14155 | | | 40.12 | 13289 | | | 31.90 | p<0.001 | 2283 | | | 40.08 | 1561 | | | 45.99 | p<0.001 |
| **Age** | | |  | | |  |  | | |  |  |  | | |  |  | | |  |  |
| 15-19 | | | 3898 | | | 35.70 | 3413 | | | 25.64 | p<0.001 | 727 | | | 34.77 | 422 | | | 33.38 | p>0.100 |
| 20-24 | | | 4451 | | | 32.68 | 3557 | | | 23.08 | p<0.001 | 643 | | | 32.44 | 391 | | | 34.19 | p>0.100 |
| 25-34 | | | 8897 | | | 34.57 | 7379 | | | 22.34 | p<0.001 | 1541 | | | 34.88 | 911 | | | 42.77 | p<0.001 |
| 35-49 | | | 11574 | | | 39.98 | 11300 | | | 32.82 | p<0.001 | 1883 | | | 41.23 | 1269 | | | 46.63 | p<0.001 |
| **Education** | | |  | | |  |  | | |  |  |  | | |  |  | | |  |  |
| No schooling | | | 4582 | | | 42.12 | 2370 | | | 35.89 | P<0.010 | 395 | | | 46.59 | 119 | | | 49.86 | p>0.100 |
| Primary | | | 3240 | | | 38.73 | 3116 | | | 33.89 | p<0.001 | 466 | | | 34.32 | 293 | | | 49.90 | p<0.001 |
| Secondary | | | 15189 | | | 36.67 | 13161 | | | 27.45 | p<0.001 | 2695 | | | 37.61 | 1650 | | | 41.98 | P<0.010 |
| Higher | | | 5809 | | | 30.88 | 7002 | | | 21.90 | p<0.001 | 1238 | | | 33.74 | 931 | | | 38.43 | p<0.050 |
| **Wealth** | | |  | | |  |  | | |  |  |  | | |  |  | | |  |  |
| Poorest | | | 829 | | | 38.78 | 938 | | | 34.55 | p>0.100 | 108 | | | 37.40 | 97 | | | 44.24 | p>0.100 |
| Poorer | | | 4036 | | | 39.53 | 3614 | | | 31.96 | p<0.001 | 599 | | | 37.67 | 393 | | | 45.62 | P<0.050 |
| Middle | | | 8021 | | | 38.07 | 6887 | | | 30.75 | p<0.001 | 1328 | | | 39.49 | 856 | | | 45.01 | P<0.010 |
| Richer | | | 9179 | | | 37.30 | 7843 | | | 26.76 | p<0.001 | 1587 | | | 37.01 | 908 | | | 41.87 | P<0.050 |
| Richest | | | 6755 | | | 31.90 | 6368 | | | 21.32 | p<0.001 | 1172 | | | 33.90 | 739 | | | 36.31 | p>0.100 |
| **Caste** | | |  | | |  |  | | |  |  |  | | |  |  | | |  |  |
| Schedule tribe | | | 509 | | | 35.37 | 546 | | | 29.58 | P<0.050 | 87 | | | 23.15 | 104 | | | 21.20 | p>0.100 |
| Schedule caste | | | 8286 | | | 41.62 | 7319 | | | 30.56 | p<0.001 | 1271 | | | 39.94 | 852 | | | 45.03 | P<0.050 |
| Other Backward Class | | | 19478 | | | 34.72 | 17241 | | | 26.38 | p<0.001 | 3388 | | | 36.44 | 2004 | | | 42.14 | P<0.001 |
| Other | | | 465 | | | 27.87 | 482 | | | 21.35 | p<0.050 | 35 | | | 29.64 | 27 | | | 28.27 | p>0.100 |
| **Religion** | | |  | | |  |  | | |  |  |  | | |  |  | | |  |  |
| Hindu | | | 26092 | | | 36.98 | 23316 | | | 28.19 | p<0.001 | 4348 | | | 38.31 | 2771 | | | 42.75 | P<0.001 |
| Muslim | | | 1328 | | | 31.35 | 902 | | | 19.15 | p<0.001 | 219 | | | 29.13 | 108 | | | 40.22 | P<0.050 |
| Others | | | 1400 | | | 34.52 | 1431 | | | 21.45 | p<0.001 | 228 | | | 20.16 | 114 | | | 24.69 | p>0.100 |
| **Marital Status** | | |  | | |  |  | | |  |  |  | | |  |  | | |  |  |
| Never married | | | 6219 | | | 34.89 | 5579 | | | 26.31 | p<0.001 | 1810 | | | 33.86 | 1159 | | | 36.79 | p<0.001 |
| Married | 21082 | | | | 36.83 | | 18472 | | 27.43 | | p<0.001 | 2953 | | 39.10 | | 1798 | | 44.93 | | P<0.001 |
| Other | | | 1519 | | | 40.37 | 1629 | | | 32.32 | p<0.001 | 31 | | | 24.62 | 36 | | | 60.29 | P<0.050 |
| Total | | | 28820 | | | 36.60 | 25650 | | | 27.50 | p<0.001 | 4794 | | | 37.03 | 2993 | | | 41.97 | P<0.001 |

| **Table S5: Percentage distribution of women and men covered by PFHIS according to background characteristics in Telangana in NFHS-4 and NFHS-5** | | | | | | | | | | | | | | | |
| --- | --- | --- | --- | --- | --- | --- | --- | --- | --- | --- | --- | --- | --- | --- | --- |
|  | **Women** | | | | | | | | | | **Men** | | | | |
|  | **NFHS-4** | | | | **NFHS-5** | | | |  | | **NFHS-4** | | **NFHS-5** | |  |
| **Background characteristics** | **N** | | **%** | | **N** | | **%** | | **p value** | | **N** | **%** | **N** | **%** | **p value** |
| **Place of Residence** |  | |  | |  | |  | |  | |  |  |  |  |  |
| Urban | 3707 | | 42.82 | | 10275 | | 45.29 | | P<0.010 | | 509 | 55.85 | 1408 | 56.51 | p>0.100 |
| Rural | 3860 | | 70.22 | | 17243 | | 68.52 | | P<0.050 | | 545 | 77.65 | 2101 | 74.50 | p>0.100 |
| **Age** |  | |  | |  | |  | |  | |  |  |  |  | p>0.100 |
| 15-19 | 1002 | | 63.42 | | 3379 | | 63.40 | | p>0.100 | | 173 | 72.60 | 532 | 65.79 | p>0.100 |
| 20-24 | 1354 | | 44.28 | | 4106 | | 49.78 | | P<0.001 | | 155 | 66.20 | 503 | 69.24 | p>0.100 |
| 25-34 | 2474 | | 52.91 | | 8520 | | 49.14 | | P<0.001 | | 339 | 61.33 | 1103 | 66.06 | p>0.100 |
| 35-49 | 2737 | | 64.07 | | 11513 | | 70.32 | | P<0.001 | | 387 | 70.11 | 1370 | 68.12 | p>0.100 |
| **Education** |  | |  | |  | |  | |  | |  |  |  |  |  |
| No schooling | 2425 | | 71.72 | | 8976 | | 73.91 | | P<0.050 | | 176 | 79.38 | 509 | 73.27 | p>0.100 |
| primary | 660 | | 64.73 | | 2007 | | 65.39 | | p>0.100 | | 98 | 71.75 | 226 | 67.54 | p>0.100 |
| secondary | 3283 | | 52.51 | | 11852 | | 54.54 | | P<0.050 | | 553 | 66.75 | 1943 | 67.55 | p>0.100 |
| higher | 1199 | | 33.96 | | 4683 | | 43.93 | | P<0.001 | | 227 | 56.55 | 830 | 62.90 | p>0.100 |
| **Wealth** |  | |  | |  | |  | |  | |  |  |  |  |  |
| poorest | 390 | | 65.86 | | 1164 | | 60.91 | | p>0.100 | | 41 | 71.81 | 139 | 61.00 | p>0.100 |
| poorer | 1171 | | 69.83 | | 4572 | | 68.75 | | p>0.100 | | 153 | 74.87 | 516 | 68.46 | p>0.100 |
| middle | 1979 | | 70.25 | | 7991 | | 69.24 | | p>0.100 | | 297 | 78.51 | 913 | 73.02 | p>0.100 |
| richer | 2244 | | 56.60 | | 7880 | | 60.62 | | P<0.001 | | 320 | 64.52 | 1233 | 72.30 | P<0.010 |
| richest | 1784 | | 31.58 | | 5911 | | 39.02 | | P<0.001 | | 243 | 50.95 | 708 | 51.50 | p>0.100 |
| **Caste** |  | |  | |  | |  | |  | |  |  |  |  |  |
| Schedule tribe | 606 | | 68.98 | | 2400 | | 61.74 | | P<0.001 | | 85 | 86.99 | 256 | 64.67 | P<0.001 |
| Schedule caste | 1391 | | 64.78 | | 6185 | | 64.72 | | p>0.100 | | 158 | 69.65 | 785 | 73.99 | p>0.100 |
| Other Backward Class | 4384 | | 57.23 | | 16103 | | 60.37 | | P<0.001 | | 664 | 67.35 | 2128 | 68.05 | p>0.100 |
| Other | 1119 | | 38.79 | | 2640 | | 44.97 | | P<0.001 | | 141 | 50.78 | 333 | 48.80 | p>0.100 |
| **Religion** |  | |  | |  | |  | |  | |  |  |  |  |  |
| Hindu | 6476 | | 58.28 | | 23635 | | 61.27 | | P<0.001 | | 901 | 67.66 | 2826 | 69.04 | p>0.100 |
| Muslim | 820 | | 44.61 | | 2965 | | 48.51 | | P<0.050 | | 126 | 68.73 | 598 | 60.68 | p>0.100 |
| Others | 272 | | 58.08 | | 918 | | 59.66 | | p>0.100 | | 27 | 41.53 | 85 | 55.28 | p>0.100 |
| **Marital Status** |  | |  | |  | |  | |  | |  |  |  |  |  |
| Never married | 1409 | | 62.29 | | 3189 | | 63.24 | | p<0.001 | | 381 | 69.46 | 1324 | 1324 | p<0.001 |
| Married | | 5605 | | 54.58 | | 20268 | | 57.83 | | P<0.001 | 661 | 65.84 | 2152 | 2152 | p>0.100 |
| Other | 553 | | 65.25 | | 2208 | | 70.61 | | p>0.100 | | 12 | 63.75 | 33 | 33 | p>0.100 |
| Total | 7567 | | 56.80 | | 27518 | | 59.85 | | P<0.001 | | 1054 | 67.12 | 3509 | 67.28 | p>0.100 |

| **Table S6: Inequality change between NFHS4 and NFHS 5** | | | | | | | | | | | | | | | | | |
| --- | --- | --- | --- | --- | --- | --- | --- | --- | --- | --- | --- | --- | --- | --- | --- | --- | --- |
|  | Residence | | Marital status | | Age | | Education | | Wealth | | Social group | | | Religion | | Overall absolute score | Overall relative score |
| **Women** | Absolute | Relative | Absolute | Relative | Absolute | Relative | Absolute | Relative | Absolute | Relative | Absolute | | Relative | Absolute | Relative |  |  |
| Andhra Pradesh | 1 | *0* | 1 |  | 1 | *1* | 1 | *1* | 1 | *1* | 1 | | *1* | 0 | *0* | 6 | 4 |
| Karnataka | 0 | 1 | 0 | 0 | 0 | 1 | 0 | 1 | 0 | 0 | 0 | | 0 | 0 | 0 | 0 | 3 |
| Kerala | *1* | 1 | 0 | 0 | *1* | 1 | *1* | 0 | *1* | 0 | *1* | | 0 | *1* | 1 | 6 | 3 |
| Tamil Nadu | 1 | *0* | 0 | 0 | 0 | *0* | 0 | *1* | 1 | *1* | 1 | | *1* | 0 | *0* | 3 | 3 |
| Telangana | 1 | 0 | 1 | 0 | 1 | 1 | 1 | 1 | 1 | 1 | 1 | | 1 | 1 | 1 | 7 | 5 |
| Interpretation | magnitudes are small | | | | | | | | | | | the increase in inequality tends to favour the poor, which may not be a bad thing. | | | | | |
| **Men** |  |  |  |  |  |  |  |  |  |  |  | |  |  |  |  |  |
| Andhra Pradesh | 1 | 1 | 1 |  | 1 | 1 | 1 | 1 | 1 | 1 | 1 | | 1 | 0 | 0 | 6 | 5 |
| Karnataka | 0 | 1 | 1 |  | 1 | 1 | 0 | 0 | 0 | 0 | 0 | | 0 | 1 | 1 | 3 | 3 |
| Kerala | 1 | 0 | 1 | 0 | 1 | 1 | 1 | 1 | 1 | 1 | 1 | | 1 | 1 | 1 | 7 | 5 |
| Tamil Nadu | 1 | 0 | 1 | 0 | 0 | 1 | 1 | 1 | 1 | 1 | 0 | | 0 | 0 | 0 | 4 | 3 |
| Telangana | 1 | 0 | 1 | 0 | 1 | 1 | 1 | 1 | 1 | 1 | 0 | | 0 | 1 | 1 | 6 | 4 |
| Interpretation | magnitudes are small | | | | | | | | | | | the increase in inequality tends to favour the poor, which may not be a bad thing. | | | | | |

Note: In the columns representing NFHS-5 summary measures of inequality, green indicates a reduction in the magnitude of inequality when compared to NFHS-4 while red indicates an increase.
